# Relationship of troponin T to cardiac MRI criteria for acute myocarditis

**DOI:** 10.1186/1532-429X-13-S1-P271

**Published:** 2011-02-02

**Authors:** Carmen P Lydell, Emmanuelle Vermes, Helene C Childs, Iacopo Carbone, Ahmed Aljizeeri, Matthias G Friedrich, Naeem Merchant

**Affiliations:** 1 Foothills Medical Centre, University of Calgary, Calgary, AB, Canada

## Objective

To determine the relationship between biochemical markers of myocardial injury in acute myocarditis and the cardiac MR imaging features.

## Background

Cardiac MR criteria for acute myocarditis (“Lake Louise Criteria”) include scarring as defined by high-signal-intensity areas in late gadolinium enhancement (LGE), and inflammatory markers as defined by an increased early contrast uptake (early gadolinium enhancement, EGE) and edema (increased signal intensity in T2 signal weighted images). Troponin is a widely used clinical marker for cardiomyocyte death; however, the relationship between biochemical markers of myocardial injury and these imaging features has not been clearly established.

## Methods

Thirty-one patients who had troponin-T levels measured at presentation and had the diagnosis of acute myocarditis based on clinical factors and CMR criteria “Lake Louise Criteria” were evaluated. MR images were assessed for the volume of LGE, %EGE and EGE ratio, as well as T2 signal intensity ratio normalized to skeletal muscle. Ordinary least squared linear regression was used to determine the relationship between these imaging features and peak serum troponin-T concentration in the acute presentation.

## Results

There was a linear increase in the total volume of LGE and EGE with increases in square root troponin-T concentration (R^2^= 0.57, Beta coefficient= 16.8, p<0.001) and (R^2^=0.33, beta coefficient 4.1, p=0.001), respectively. The T2 signal intensity ratio and EGE ratio did not show a relationship to troponin levels (R^2^=0.08, Beta coefficient 0.05, p=0.114) and (R^2^=0.00, Beta coefficient -0.12, p=0.80), respectively.

## Conclusions

In the setting of acute myocarditis troponin-T concentrations show the strongest correlation with LGE and, to a lesser degree with EGE values. There is no correlation to the calculated EGE ratio or T2 ratio. These finding suggest that LGE specifically reflects irreversible myocardial injury, whereas other CMR criteria appear to reflect processes that are not associated with myocardial necrosis.

